
JOURNAL INFORMATION
==============================
NLM Title Abbreviation: CPT Pharmacometrics Syst Pharmacol
Journal ID: CPT Pharmacometrics Syst Pharmacol
NLM Journal ID: 101580011
Journal ID: PSP4

CPT: Pharmacometrics & Systems Pharmacology

EISSN: 2163-8306
Publisher: Wiley

ARTICLE INFORMATION
==============================
PMCID: PMC11474808
DOI: 10.1002/psp4.12823
Article ID: PSP412823
Article version: 1
Subjects: Issue Information, Issue Information

Issue Information

Electronic publication date: 2023 Aug 16
Publication date: 2023 Aug
Volume: 12
Issue: 8
First page: 1043
Last page: 1046
Copyright: © 2023 The Authors. CPT: Pharmacometrics & Systems Pharmacology published by Wiley Periodicals LLC on behalf of American Society for Clinical Pharmacology and Therapeutics.
License: This is an open access article under the terms of the http://creativecommons.org/licenses/by/4.0/ License, which permits use, distribution and reproduction in any medium, provided the original work is properly cited.
License URL: https://creativecommons.org/licenses/by/4.0/

Figure count: 0
Table count: 0
Page count: 3

source-schema-version-number: 2.0
cover-date: August 2023
details-of-publishers-convertor: Converter:WILEY_ML3GV2_TO_JATSPMC version:6.4.9 mode:remove_FC converted:15.10.2024

==============================

An Official Journal of ASCPT and ISoP

### CPT: Pharmacometrics & Systems Pharmacology

Open Access

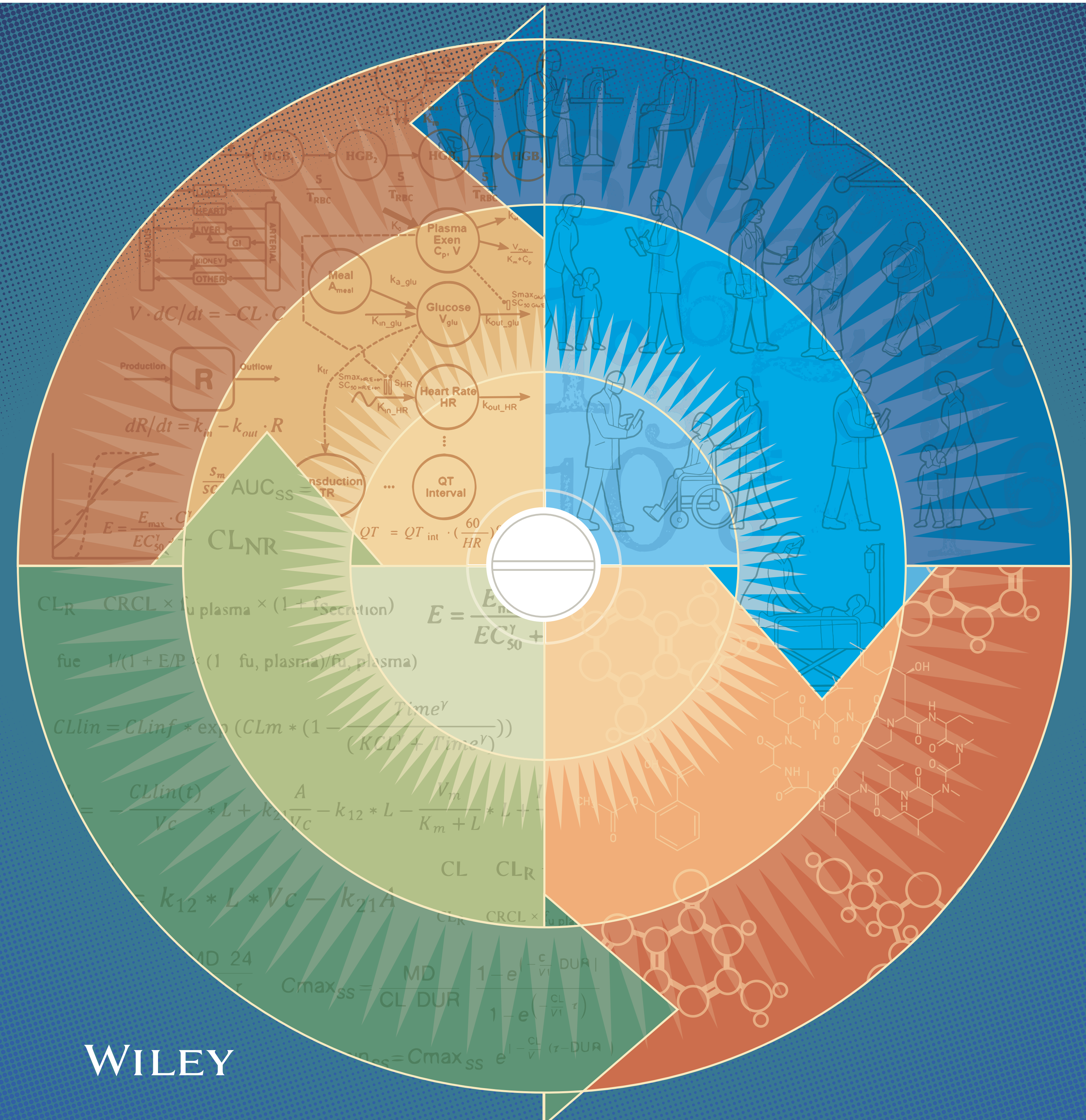

WILEY

### CPT: Pharmacometrics & Systems Pharmacology

WILEY

### Editor-in-Chief

France Mentré, MD, PhD, *School of Medicine of University Paris Diderot, Paris, France*

### Deputy Editor-in-Chief

Karen Rowland Yeo, PhD, *Certara UK Ltd., Sheffield, UK*

### Associate Editors

Jonathan French, ScD, *Metrum Research Group, Tariffville, CT, USA*
Stefanie Hennig, MSc, PhD, *Certara Strategic Consulting, Brisbane, Australia*
Sriram Krishnaswami, PhD, *Pfizer, Groton, CT, USA*
Vikram Sinha, PhD, *Novartis Institute of Biomedical Research, Berwyn, PA, USA*
Ping Zhao, PhD, *Bill & Melinda Gates Foundation, Seattle, WA, USA*
Eric Sobie, PhD, *Metrum Research Group, Tariffville, CT, USA*

### Editors-in-Training

Rajat Desikan, PhD, *GSK, UK, Middlesex, UK*
Priya Jayachandran, PharmD, *Regeneron Pharmaceuticals, Inc., San Francisco, CA, USA*

### American Society for Clinical Pharmacology and Therapeutics

***Chief Executive Officer***
Abigail W. Gorman, *Alexandria, VA*
***Chief Operating Officer***
Elise S. Laffman-Johnson, *Alexandria, VA*
***Managing Editor***
Alethea B. Gerding, *Alexandria, VA*
***Deputy Managing Editor***
Alaina Webster, *Alexandria, VA*
***Senior Editorial Coordinator***
Aimee Greene, *Alexandria, VA*

### Editorial Board

Antoinette Ajavon-Hartmann, PhD, *Bristol Myers Squibb, Lawrence Township, NJ, USA*
Vivaswath S. Ayyar, PhD, *Janssen Research & Development, Spring House, PA*
Robert Bies, PharmD, PhD, *State University of New York at Buffalo, Buffalo, NY, USA*
Marc Birtwistle, PhD, *Clemson University Clemson, SC, USA*
Marylore Chenel, PharmD, PhD, *Pharmetheus Uppsala, Sweden*
Emmanuel Chigutsa, PhD, *Eli Lilly and Company, Indianapolis, IN, USA*
Teresa Dalla Costa, PhD, *Federal University of Rio Grande do Sul - UFRGS Porto Alegre, Brazil*
Paolo Denti, PhD, *University of Cape Town Cape Town, South Africa*
Narendra M Dixit, PhD, *Indian Institute of Science, Bengaluru, India*
James M. Gallo, PharmD, PhD, *University at Buffalo, Buffalo, NY, USA*
Wei Gao, PhD, *EMD Serono, Billerica, MA, USA*
Manish Gupta, PhD, FCP, *GenMab; Princeton, NJ, USA*
Bart Hendriks, PhD, *Novartis Institutes for BioMedical Research Cambridge, MA, USA*
Nick Holford, MBChB, *The University of Auckland Auckland, New Zealand*
Brett A. Howell, PhD, *DILIsym Services Division, Simulations Plus, Inc., Research Triangle Park, NC, USA*
Manuel Ibarra, PhD, *Universidad de la República, Montevideo, Uruguay*
Siv Jönsson, PhD, *Uppsala University Sweden, Uppsala, Sweden*
Kristin Karlsson, PhD, *Uppsala University Uppsala, Sweden*
Helen Kastrissios, PhD, *QuanTx Consulting, Brisbane, Australia*
Ron Keizer, PhD, *InsightRX San Francisco, CA, USA*
Steven E. Kern, PhD, *Global Health Labs, Bellevue, WA, USA*
Andrzej Kierzek, PhD, *Certara QSP; Sheffield, United Kingdom*
Qi Liu, PhD, *US Food and Drug Administration, Silver Spring, MD*
James Lu, PhD, *Genentech, Inc., South San Francisco, CA, USA*
Rajanikanth (Raj) Madabushi, PhD, *US Food and Drug Administration, Silver Spring, MD*
Efthymios Manolis, MSc, *European Medicines Agency London, United Kingdom*
Jackson K. Mukonzo, PhD, *Makerere University, Kampala Uganda*
Joakim Nyberg, PhD, *Pharmetheus, Uppsala, Sweden*
Elsje Pienaar, PhD, *Weldon School of Biomedical Engineering, Purdue University, West Lafayette, IN, USA*
Daniel Scholes Rosenbloom, PhD, *Merck & Co., Rahway, NJ, USA*
Joe Standing, PhD, *University College London London, United Kingdom*
Natasha Strydom, PhD, *Amgen, Inc., San Francisco, CA, USA*
Yuichi Sugiyama, PhD, *Riken Innovation Center Sugiyama Laboratory Yokohama, Japan*
Joel Tarning, PhD, *Mahidol-Oxford Tropical Medicine Research Unit Bangkok, Thailand*
Michael Tortorici, PharmD, PhD, *Aro Biotherapeutics, Philadelphia, PA, USA*
Coen van Hasselt, PhD, *Leiden University, Leiden, the Netherlands*
Paolo Vicini, PhD, MBA, *Confo Therapeutics, Ghent, Belgium*
Camille Vong, PhD, *Novartis AG Pharma, Basel, Switzerland*
Xinyuan (Susie) Zhang, PhD, *Daiichi Sankyo, Inc. Basking Ridge, NJ, USA*

### Overview

*CPT: Pharmacometrics & Systems Pharmacology* (PSP) is an official open access journal of the American Society for Clinical Pharmacology and Therapeutics (ASCPT) and International Society of Pharmacometrics (ISoP).

*CPT: Pharmacometrics & Systems Pharmacology* is a cross-disciplinary journal devoted to publishing advances in quantitative (e.g., modeling and simulation) methods as applied in pharmacology, physiology and therapeutics in humans. The journal welcomes original research articles, reviews and tutorials that bridge the following areas: pharmacometrics, modeling and simulation as applied to the design and evaluation of clinical trials, systems pharmacology modeling, particularly with a mechanistic link to human (patho)physiology, disease modeling, "population" or mixed-effects pharmacokinetics and pharmacodynamics (PKPD) modeling, modeling and simulation to support translational research, physiologically-based pharmacokinetics (PBPK), model-based meta-analyses of clinical trials, mechanism-based pharmacokinetic-pharmacodynamic modeling, computational pharmacology, bioinformatics, comparative efficacy, effectiveness and cost-effectiveness. Systems pharmacology may involve the application of systems biology approaches to study drug activities, targets and effects. The discipline is often defined with reference to engineering and pharmacological principles as the quantitative analysis of the dynamic interactions between drugs and a biologic system that aims to understand the behavior of the system as a whole. The common focus will be on quantitative methods that improve our understanding of pharmacology and therapeutics in humans.

**Abstracting and Indexing**
- Embase (Elsevier)
- SCOPUS (Elsevier)
- MEDLINE/PubMed indexing

**Society Information**

The American Society for Clinical Pharmacology and Therapeutics (ASCPT) was founded in 1900, and consists of over 2,200 professionals whose primary interest is to advance the science and practice of clinical pharmacology and translational medicine for the therapeutic benefit of patients and society. ASCPT is the largest scientific and professional organization serving the disciplines of Clinical Pharmacology and Translational Medicine.

**All articles published in this journal are freely available to read, download, and share. Articles are published under the terms of the Creative Commons Attribution Non-Commercial License which permits use, distribution, and reproduction in any medium, provided the original work is properly cited and is not used for commercial purposes.**

**Disclaimer**

The Publisher, the American Society for Clinical Pharmacology and Therapeutics, the International Society of Pharmacometrics, and Editors cannot be held responsible for errors or any consequences arising from the use of information contained in this journal; the views and opinions expressed do not necessarily reflect those of the Publisher, the American Society for Clinical Pharmacology and Therapeutics, the International Society of Pharmacometrics or Editors, neither does the publication of advertisements constitute any endorsement by the Publisher, the American Society for Clinical Pharmacology and Therapeutics, the International Society of Pharmacometrics or Editors of the products advertised.

**Publisher**

*CPT: Pharmacometrics & Systems Pharmacology* is published by Wiley Periodicals, LLC, 101 Station Landing, Suite 300, Medford, MA 02155; Telephone: 781-388-8200; Fax: 781-388-8210.

VOLUME 12 NUMBER 8 | AUGUST 2023

CONTENTS

CPT: Pharmacometrics &
Systems Pharmacology

### TUTORIAL

### TUTORIAL

**1047** Using machine learning surrogate modeling for faster QSP VP cohort generation
**Renée C. Myers, Florian Augustin, Jérémy Huard and Christina M. Friedrich**

### RESEARCH

### ARTICLES

**1060** Population pharmacokinetic dosimetry model using imaging data to assess variability in pharmacokinetics of $^{177}$Lu-PSMA-617 in prostate cancer patients
**Hinke Siebinga, Bastiaan M. Privé, Steffie M. B. Peters, James Nagarajah, Thomas P. C. Dorlo, Alwin D. R. Huitema, Berlinda J. de Wit-van der Veen and Jeroen J. M. A. Hendrikx**

**1072** Knowledge graph aids comprehensive explanation of drug and chemical toxicity
**Yun Hao, Joseph D. Romano and Jason H. Moore**

**1080** Population pharmacokinetic analysis of sparsentan in healthy volunteers and patients with focal segmental glomerulosclerosis
**Russell Wada, Huub Jan Kleijn, Lu Zhang and Shang-Chiung Chen**

**1093** Physiologically-based pharmacokinetic pharmacodynamic parent-metabolite model of edoxaban to predict drug–drug-disease interactions: M4 contribution
**Ruijuan Xu, Wenyuan Liu, Weihong Ge, Hua He and Qing Jiang**

**1107** Population pharmacokinetic analysis of the P2X3-receptor antagonist gefapixant
**Akshita Chawla, Anna Largajolli, Azher Hussain, Huub Kleijn, Sihem Ait-Oudhia, Judith Anton, Hari Krishna Ananthula, Jesse Nussbaum, Carmen La Rosa and Ferdous Gheyas**

**1119** Combination of antidepressants and antipsychotics as a novel treatment option for psychosis in Alzheimer's disease
**Peihao Fan, Lang Zeng, Ying Ding, Julia Kofler, Jonathan Silverstein, Joshua Krivinko, Robert A. Sweet and Lirong Wang**

**1132** Model-based meta-analysis of ethnic differences and their variabilities in clearance of oral drugs classified by clearance mechanism
**Hiromi Sato, Rika Marutani, Ryota Takaoka, Daniel Mori-Fegan, Xinying Wang, Kazuya Maeda, Hiroyuki Kusuhara, Hiroshi Suzuki, Hideki Yoshioka and Akihiro Hisaka**

**1143** Physiologically-based pharmacokinetic modeling of quinidine to establish a CYP3A4, P-gp, and CYP2D6 drug–drug–gene interaction network
**Denise Feick, Simeon Rüdesheim, Fatima Zahra Marok, Dominik Selzer, Helena Leonie Hanae Loer, Donato Teutonico, Sebastian Frechen, Maaike van der Lee, Dirk Jan A. R. Moes, Jesse J. Swen, Matthias Schwab and Thorsten Lehr**

**1157** Population pharmacokinetics of atacicept in systemic lupus erythematosus: An analysis of three clinical trials
**Maria Pitsiu, Özkan Yalkinoglu, Colm Farrell, Pascal Girard, Cristina Vazquez-Mateo and Orestis Papasouliotis**

**1170** Modeling tumor size dynamics based on real-world electronic health records and image data in advanced melanoma patients receiving immunotherapy
**Perrine Courlet, Daniel Abler, Monia Guidi, Pascal Girard, Federico Amato, Naik Vietti Violi, Matthieu Dietz, Nicolas Guignard, Alexandre Wicky, Sofiya Latifyan, Rita De Micheli, Mario Jreige, Clarisse Dromain, Chantal Csajka, John O. Prior, Karthik Venkatakrishnan, Olivier Michielin, Michel A. Cuendet and Nadia Terranova**

**1182 CORRIGENDUM**
